# Editorial: Omics in seed development: challenges and opportunities for improving of seed quality and yield in model and crop plants

**DOI:** 10.3389/fpls.2025.1568039

**Published:** 2025-02-20

**Authors:** Changye Yang, Zhaorong Hu, Raju Datla, Daoquan Xiang

**Affiliations:** ^1^ Aquatic and Crop Resource Development, National Research Council Canada, Saskatoon, SK, Canada; ^2^ Frontiers Science Center for Molecular Design Breeding, Key Laboratory of Crop Heterosis and Utilization, Beijing Key Laboratory of Crop Genetic Improvement, China Agricultural University, Beijing, China; ^3^ Global Institute for Food Security, University of Saskatchewan, Saskatoon, SK, Canada

**Keywords:** seed development, omics, genomics, transcriptomics, proteomics, metabolomics

## Introduction

Seeds are a fundamental component in the life cycle of sexually reproducing plants, marking both the beginning of a new generation through germination and the culmination of the reproductive phase through seed production. Plant species exhibit remarkable diversity in seed characteristics, particularly in size, number, and composition, with seeds developing through precisely coordinated programs across embryo, endosperm, and seed coat compartments. In crop plants, seeds represent the most economically significant products, directly influencing both crop quality and yield. Recent advances in omics technologies including genomics, transcriptomics, proteomics, and metabolomics have dramatically enhanced our understanding of seed biology (Liu et al., 2022). These comprehensive findings have provided unprecedented insights into the genetic and molecular mechanisms underlying seed development, germination, and composition (Chen et al., 2023; Yu et al., 2023; Klcova et al., 2024). Seed omics studies have facilitated the identification of key genes and pathways associated with essential traits, contributing to the development of advanced breeding strategies that improve desirable attributes such as nutritional content, fertilization efficiency, and yield (Yuan et al., 2024). Moreover, these studies play a crucial role in optimizing seed quality and enhancing crop resilience to various environmental and climatic challenges. As global food demand continues to rise, insights from seed omics research have become increasingly vital for achieving sustainable agriculture and food security goals.

This Research Topic focuses on recent advances in seed omics research, comprising twelve articles that explore diverse aspects of the field. Five review articles examine recent progress in seed omics, while seven research articles present findings on omics data processing, new methodological developments, and seed trait analysis, as detailed below.

## New technology and methods

Several articles in this Research Topic have developed new approaches in seed structural imaging, machine learning detection, and breeding procedures. The study by Ashe et al., presented a suite of Synchrotron radiation (SR) related imaging methodologies for applications in research studies using plants seeds. The datasets generated from this study represent diverse plant species that include *Citrullus* sp. (watermelon), *Brassica* sp. (canola), *Pisum sativum* (pea), and *Triticum durum* (wheat). The authors have introduced the SR micro-computed tomography (SR-µCT) non-destructive imaging method, with advanced capabilities for unveiling detailed internal seed microstructures and their three-dimensional morphologies. Additionally, presented methods for using synchrotron X-rays, including X-ray absorption spectroscopy (XAS) and X-ray fluorescence (XRF) imaging to reveal elemental structural distributions that allow the spatial mapping of micronutrients in seed sub-compartments to determine their speciation characteristics.

Once the large omics datasets are acquired, the researchers focus on processing technologies. In this context, a deep learning network SGR-YOLO, proposed by Yao et al., specifically designed to detect seed germination rates in wild rice. The backbone of the network is based on YOLOv7 with the addition of a bi-directional feature pyramid network (BiFPN) and ECA lightweight attention mechanism. The trained model presented in this study will facilitate processing of images of wild rice grains in hydroponic boxes and Petri dishes, outputting bounding boxes that identify each grain, irrespective of germination status.

Moreover, Song et al. employed a non-GMO breeding approach to reduce food allergen *Len c3* in *Lens culinaris* seeds. The study, first identified a lipid transfer protein (LTP) gene *Lcu.2RBY.4g013600* that encodes the lentil allergen *Len c3*. The authors of this study introduced gene screening to search for natural mutations of the *Len c3* allergen-encoding gene for mitigation of food allergen *Len c3*. This research resulted in the selection of lentil hybrids with reduced allergenic traits.

## Reviews on current advancements

Nearly every important trait of seeds, such as nutritional content, is regulated by a multitude of factors. For instance, Nosworthy et al. reviewed studies related to the protein content and quality of pulses protein crops, reflecting the growing interest in the development of plant-based proteins. This review highlighted the quality of pulse proteins is likely determined by the Protein Efficiency Ratio and Protein Digestibility Corrected Amino Acid Score as well as bioactive properties of specific bioactive peptides related to amelioration of hypertension and diabetes. In another review, Yuan et al. explored the diverse roles of transcription factors (TFs) during zygotic embryogenesis (ZE) and somatic embryogenesis (SE). This review focused on the master TFs involved in embryogenesis, such as *BABY BOOM* (*BBM*) from the *APETALA2*/Ethylene-Responsive Factor (*AP2*/*ERF*) family, *WUSCHEL* and *WUSCHEL*-related homeobox (*WOX*) from the homeobox family, etc. This review also covered several plant species and discussed future perspectives on the role of transcription factors in plant zygotic embryogenesis. Furthermore, Go et al. from the University of British Columbia reviewed the role of transcriptional activators and repressors in epigenetic repression of the embryonic and seed maturation programs in seedlings. Their review illustrates the roles of core proteins and accessory components in the epigenetic machinery, as well as the similarities in the activation and repression of the embryonic and seed maturation programs.

Extensive research has also been conducted on generating various omics data for seed research. Wang et al. summarized the achievements in peanut seed development regulation and trait analysis based on reference genome-guided omics studies. Following the completion of the peanut reference genome, numerous studies have utilized omics data to elucidate the quantitative trait loci (QTL) associated with seed weight, oil content, and protein content. This review highlights the significant progresses in the understanding of molecular basis of peanut seed development and demonstrates the importance of omics data in elucidating the regulatory mechanisms involved. In another review, Ji et al. summarized the recent studies using the application of omics technologies such as genomics, transcriptomics, proteomics and metabolomics. These studies shed light on the mechanisms underlying seed development in wheat and maize, representing the C3 and C4 plants in *Poaceae* family. The *Poaceae* family, commonly known as the grass family, due to its diverse range of species, represents economically the most important crops for human society. The diversity represented in these species also offers photosynthetic pathways of C3 and C4 plants which introduces intriguing variations in their physiological and biochemical processes, potentially affecting their respective seed developmental programs.

## Seed omics data processing

In this Research Topic, several studies also presented their research on analyzing seed behavior using omics data. Huang et al. conducted transcriptome analysis of differential sugar accumulation in the embryos of *Castanea mollissima* (Chinese chestnut). This study utilized the metabolomic and transcriptomic datasets to investigate metabolites and genes related to sugar in two Chinese chestnut cultivars representing the high and low sugar variants. The conclusion is that the high-sugar cultivar promoted the conversion of starch to sucrose, partly due to a strong increase of the activity of the SUS-synthetic enzyme. Beyond Chinese chestnuts, a different group of researchers studied the nutritional quality of different ploidy rice using their metabolomics and transcriptomics data. In this study Xian et al. tested two different types of rice, autotetraploid rice (AJNT-4x) and diploid rice (AJNT-2x), at various time points during the seed’s endosperm development. Their results established new pathways linking gene expression to key metabolites.

Two genome-wide studies have also been conducted on different plant species. One of the studies, conducted by Chen et al. linked U-box E3 ubiquitin ligase gene family to bacterial wilt resistance in tobacco (*Nicotiana tabacum L.*) and eggplant (*Solanum melongena L.*). A total of 116 NtU-box genes from tobacco and 56 SmU-box genes from eggplant were identified in the genome, which were also categorized into five subfamilies. In addition, phylogenetic analysis also suggested a shared ancestor predating the divergence of six species (tobacco, eggplant, potato, tomato, *Arabidopsis thaliana*, and pepper). In the other study, Zhang et al. employed genomics and genetics approach to identify the genetic regulatory network for lignin content in *Brassica napus* seeds using network-based systems that integrate genotype, phenotype, and molecular phenotypes, four QTLs, eighty-three subnetworks, and three modules with 910 genes have been shown to be associated with lignin content.

## Conclusions

Altogether, the studies within this Research Topic have showcased innovative techniques in seed research, analyzed seed omics data, and also reviewed current advancements in the field. The progress in seed research methodologies and the analysis of omics data provide comprehensive insights into seed development, germination, and nutritional content. These advancements will assist researchers to identify key proteins, metabolic pathways, and genes associated with desirable seed traits, paving the way for the development of more nutritious and higher yielding seed varieties. With ongoing research and interdisciplinary collaboration, seed omics datasets hold great potential for driving further future innovations in the field.
